# 

**Published:** 1897-11

**Authors:** 


### CONTENTS:

### SELECTIONS.

Article I.—Practical Things with Illustrations. By Prof. J. G. Templeton, ... 289
" II.—The Preparation of Dental Alloys and Cements. By Louis Shaw, M. D ... 299
" III.—The Physiology of Cataphoresis. By Weston A. Price. 303
" IV.—Oxychloride of Zinc. By S. T. Kirk, D. D. S ... 308
" V.—Singular Death from Chloroform at Yardley ... 310
" VI.—The National Association of Dental Examiners ... 312
" VII.—The National Association of Dental Faculties ... 317
" VIII.—Electricity and Dentistry. By James L. Gethins ... 325

### OBITUARY.

Report of the Committee on Necrology of the National Association of Dental Faculties, at Old Point Comfort ... 329

### BIBLIOGRAPHICAL.

The American Text Book of Prosthetic Dentistry ... 326
A Manual of the Injuries and Surgical Diseases of the Mouth, Face and Jaws ... 331

### MONTHLY SUMMARY.

The Arrest of Hemorrhage ... 332

Peculiarities of Woman ... 332

Don't Use Plain Teeth ... 333

Sins ... 333

The New Dental Law in Pennsylvania ... 334

Lactic Acid in Pyorrhœa ... 334

The Dental Student ... 334

A New Wart Cure ... 335

Diamond Drills, How to Use ... 336

To Keep Spittoon Clean ... 336

